# Fabrication of SiO_x_-G/PAA-PANi/Graphene Composite With Special Cross-Doped Conductive Hydrogels as Anode Materials for Lithium Ion Batteries

**DOI:** 10.3389/fchem.2020.00096

**Published:** 2020-02-21

**Authors:** Yuanhong Liao, Kang Liang, Yurong Ren, Xiaobing Huang

**Affiliations:** ^1^School of Materials Science and Engineering, Jiangsu Collaborative Innovation Center of Photovoltaic Science and Engineering, Changzhou University, Changzhou, China; ^2^Hunan Province Cooperative Innovation Center for the Construction & Development of Dongting Lake Ecological Economic Zone, College of Chemistry and Materials Engineering, Hunan University of Arts and Science, Changde, China

**Keywords:** lithium-ion batteries, anode material, SiO_x_, PAA-PANi, graphene, conductive hydrogel

## Abstract

Silicon oxides (SiO_x_) have been considered to be the likeliest material to substitute graphite anode for lithium-ion batteries (LIBs) due to its high theoretical capacity, appropriate working potential plus rich abundance. Nevertheless, the two inherent disadvantages of volume expansion and low electrical conductivity of SiO_x_ have been a main obstacle to its application. Here, SiO_x_-G/PAA-PANi/graphene composite has been successfully synthesized by *in-situ* polymerization, in which SiO_x_-G particles linked together by a graphene-doped polyacrylic acid-polyaniline conductive flexible hydrogel and SiO_x_-G is encapsulated inside the conductive hydrogel. We demonstrate that SiO_x_-G/PAA-PANi/graphene composite possesses a discharge-specific capacity of 842.3 mA h g^−1^ at a current density of 500 mA g^−1^ after a cycle life of 100 cycles, and a good initial coulombic efficiency (ICE) of 74.77%. The superior performance probably due to the lithium ion transmission rate and the electric conductivity enhanced by the three-dimensional (3D) structured conductive polymer hydrogel.

## Introduction

Lithium-ion batteries (LIBs), presumably among the most prospective devices for energy storage, are featured with higher energy density, longer cycle life, lower self-discharging, and more safety. Developing advanced LIBs with highly advanced energy density and cyclability is an immediate need for lightweight electronics and range expansion of electric vehicles. Yet the current graphite anode with an unsatisfactory specific capacity of ~372 mA h g^−1^ (LiC_6_) can′t follow the development of modern equipment for high energy storage system (Casimir et al., 2016; Zuo et al., 2017; Han et al., 2018; Yi et al., 2018, 2019; Zheng et al., 2018, 2020; Xiao et al., 2019). Hence, silicon has become a potential candidate to replace commercial graphite anode for LIBs in that it has higher capacity (~4,200 mA h g^−1^), suitable discharge platform (~0.4 V vs. Li/Li^+^) and sufficient resources (Casimir et al., 2016; Jiang et al., 2016; Xu et al., 2018; An et al., 2019; Liu Y. et al., 2019a; Yang et al., 2019; Zhou et al., 2019; Zuo et al., 2019). Nevertheless, the two stubborn disadvantages of silicon, including the deterioration of electrode structure integrity as a result of gradual enhancement of pulverization happening in the repetition of discharge/charge process, as well as poor conductivity, have been the main obstacles to its application (Zhao et al., 2016; Zuo et al., 2017). To address the above-mentioned key issues, a series of countermeasures have been taken, such as optimizing structure, doping and coating with carbon or other conductive materials.

Compared with Si-based materials, SiO_x_-based anodes are prone to achieve remarkable electrochemical performance due to the formation of Li_2_O and Li silicates, which can form the stable solid electrolyte interphase (SEI) layer and adapt to the volume expansion of SiO_x_ during the insertion of Li^+^ (Nguyen et al., 2013; Xu et al., 2017; Liu D. et al., 2019; Liu Y. et al., 2019b; Zheng et al., 2019). Although the capacity of SiO_x_ is high, the volume multiplication usually causes this material to crack and pulverize. Besides, the low intrinsic conductivity of SiO_x_ would lead to poor rate performance (Xu et al., 2017; Xiao et al., 2018; Fang et al., 2019; Li et al., 2019; Wang et al., 2020). Different ways have been adopted to overcome these issues, including doping the host framework with conductive particle, coating the electrodes with buffer materials, bettering the morphology, cutting the size of dimension and applying more effective binders. For example, Yu et al. obtained the pomegranate-liked nano-scale SiO_x_-C by spray drying, the prepared composite presents a discharge specific capacity of 1,024 mA h g^−1^ when the current density is 500 mA g^−1^ after 200 cycles (Yu et al., 2018). Jiang et al. taken advantage of graphene bubbles to encapsulate the SiO_x_ inside to accelerate ion transmission rate of the material, achieving 80% capacity retention after 1,000 cycles (Jiang et al., 2016).

Conducting polymers (CPs), such as polyaniline (PANi), polyacetylene, polythiophene (PTh), Polypyrrole (PPy), and et al., providing special 3D network nanostructures and high conductivity as a result of their large conjugated π bonds structure (Li et al., 2009; Li J. et al., 2018; Li P. et al., 2018). At the same time, CPs have been verified as significant materials for the advancement of modern society, consisting of energy storage, semiconductor sensors and catalysis. For example, a PANi/CNT composite electrode synthesized via the way of *in-situ* chemical polymerization of aniline in a well-dispersed CNT solution revealed excellent electrochemical behavior. As the cathode material for LIBs, the PANi/CNT possessed a high energy density of 86 mA h g^−1^ at the 80th cycle and an average coulombic efficiency of 98% (Li et al., 2009). Also, SiO_x_-PANi–Ag composite electrodes were synthesized by Zhang et al. via *in-situ* polymerization, and exhibited great cycling performances (with a reversible capacity of 1,149 mA h g^−1^ after 100 cycles) (Wang et al., 2014). Wu et al. prepared SiNP-PANi with a capacity retention rate of more than 90% after 5,000 cycles at a current density of 6.0 A g^−1^ (Wu et al., 2013).

Besides, the carbon bonds of graphene are sp2 hybridized, exhibiting a number of intriguing and unique properties such as high surface area, admirable electronic conductivity and superior mechanical properties (Huang et al., 2011; Li P. et al., 2018). It is very meaningful that the properties make graphene-based materials useful for modifying silicon-based materials (Jiang et al., 2016). For example, Zhu et al. constructed Si@SiO_x_/GH composite with a stable storage capacity of 1,020 mA h g^−1^ at 4 A g^−1^ (Zhu et al., 2019).

**Scheme 1:**
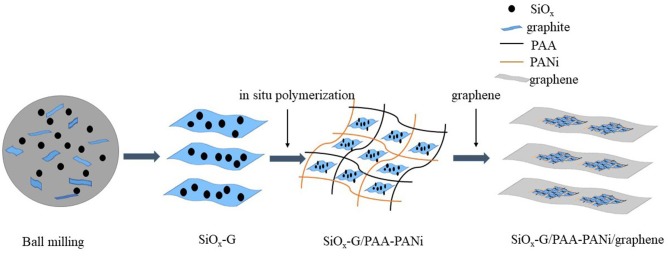
Schematic illustration for preparation of SiO_x_-G/PAA-PANi/graphene composites.

Herein, we provide a flexible and harmless method for synthesizing SiO_x_-G/PAA-PANi/graphene (Scheme 1). Firstly, SiO_x_-G was synthesized by a method of high-energy mechanical ball milling (Jiang et al., 2016). Then, Polyacrylic acid and Polyaniline are doped with each other by *in-situ* polymerization as a framework to synthesize SiO_x_-G/PAA-PANi. Finally, the graphene dispersion is added to obtain the SiO_x_-G/PAA-PANi/graphene. This intertwined doping not only can provide a continuous path for electron conduction, but also stabilize the material structure. Hence, the SiO_x_-G/PAA-PANi/graphene exhibits superior electrochemical performance.

## Experimental

### Material Preparation

#### Synthesis of SiO_x_-G

SiO_x_-G was obtained by a solid state method. Firstly, SiO_x_ is prepared by ball milling (QM-3SP4, Nanjing, China) SiO in Ar for 6 h (500 rpm, the ball-to-particulate weight ratio of 30:1). Secondly, 6.0 g of Graphite was heated at 600°C for 20 min in Ar flow and then mixed with 6.0 g of SiO_x_ by ball milling for 6 h under the same ball milling conditions as the process for preparation of SiO_x_. The above composite material is recorded as SiO_x_-G.

#### Synthesis of SiO_x_-G/PAA-PANi

Firstly, 0.005 g of PAA was weighed and dissolved in a 25 ml beaker and placed in an oven at 60°C for 1 h, and then an appropriate amount of 0.5 M NaOH was added. Secondly, 0.2 g of SiO_x_-G was dissolved in the above sodium polyacrylate solution, and ultrasonicated for 1 h, followed by stirring in an ice bath (Li P. et al., 2018). Subsequently, aniline and (NH_4_)_2_S_2_O_8_ were added and stirred for 40 min. The target production was obtained after standing, dialysis, and lyophilization.

#### Synthesis of SiO_x_-G/PAA-PANi/Graphene

The preparation steps are the same as the synthesis process of SiO_x_-G/PAA-PANi, except that the dispersion of 0.002 g of graphene is stirred for 1 h before standing. The obtained sample was named as SiO_x_-G/PAA-PANi/graphene.

### Material Characterization

The as-prepared product was characteristic of X-ray diffraction (XRD, D/max 2500 PC) with the use of Cu Ka radiation. X-ray photoelectron spectroscopy (XPS) data was recorded by the Electronic detection system (Thermo VG Scientific ESCA Lab 250). Thermogravimetric analysis (TGA) data is recorded from indoor temperature to 800°C at a rate of 10°C min^−1^ under an oxygen atmosphere. The data analysis of Fourier transform infrared (FT-IR) spectroscopy was undertaken by an IR spectrophotometer (Thermo Fisher, American). The morphology and element distribution are presented by high-resolution field emission scanning electron microscopy (FESEM, Zeiss, Germany). Transmission electron microscopy (TEM) were performed on a JEOL 2100 worked at 200 kV.

### Electrochemical Measurements

In preparation for the working electrode, the active material (75%), the conductive agent (Acetylene black,10%) and the binder (Sodium alginate,15%) were uniformly mixed to obtain the slurry, and coated on the upper part of the current collector (copper foil) and placed in a vacuum oven at 105°C for 8 h. CR2032-type coin half-cell was converged in a glove box filled with high purity argon (O_2_ and H_2_O < 0.5 ppm), with 1 M LiPF_6_ dissolved in ethylene carbonate: dimethyl carbonate: ethyl methyl carbonate (1:1:1 in volume), and 10% of fluorinated ethylene carbonate was further added as the electrolyte, Celgard 2500 film as the separator, and lithium foil as the counter electrode. The cyclic stability test and rate performance test of the material can be achieved on Land-CT 2001A instrument where the potential is 0.01 V−3 V and the current density is 500 mA g^−1^ at 25°C. Cyclic Voltammetry (CV) was recorded by electrochemical analyzer (CHI 604E, Chen He Instruments, Shanghai).

## Results and Discussion

**Figure 1:**
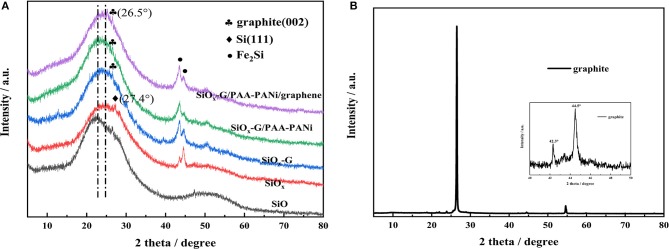
**(A)** XRD image of SiO, SiO_x_, SiO_x_-G, SiO_x_-G/PAA-PANi, and SiO_x_-G/PAA-PANi/graphene; **(B)** XRD image of graphite.

XRD patterns of four samples were compared in Figure 1A. As illustrated, commercial silicon monoxide is amorphous, which is consistent with previous findings (Hwa et al., 2013; Yu et al., 2013). Compared with SiO, the position of the broad peak of SiO_x_ shifted from 22° to 24.8°. Also, it is observed that there is a distinct characteristic peak of Si (111) located at 27.2° in as-obtained SiO_x_ sample, which might be due to that a part of SiO is reduced to Si after ball milling (Jiang et al., 2016). In addition, two peaks at 43.4° and 44.5° were observed in SiO_x_ samples, corresponding to the characteristic peak of Fe_2_Si, which might be created from the stainless steel ball and Si during the process of ball milling. Similar phenomenon was also demonstrated by Qian et al. (2017). Figure 1B shows the XRD pattern of graphite. The crystal plane characteristic peak of graphite (002) is situated at 26.5° in SiO_x_-G, SiO_x_-G/PAA-PANi and SiO_x_-G/PAA-PANi/graphene samples (Figure 1A), showing that the existence of graphite in the three samples.

**Figure 2:**
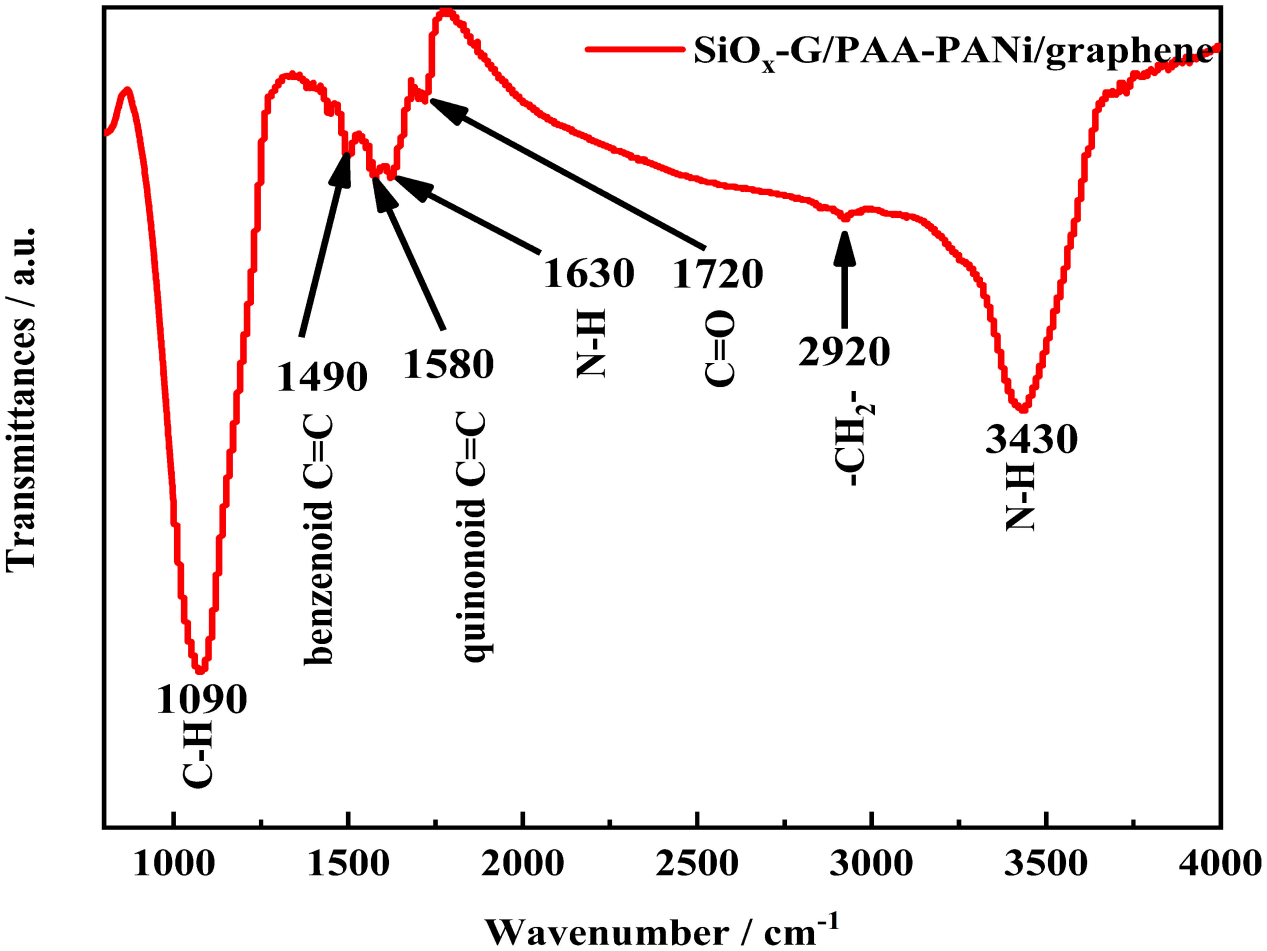
FT-IR image of SiO_x_-G/PAA-PANi/graphene.

Fourier transform infrared (FT-IR) spectrum of SiO_x_-G/PAA-PANi/graphene is shown in Figure 2. The absorption peaks at 3,430 and 2,920 cm^−1^ correspond to the N-H bending vibration absorption peak and -CH_2_- stretching vibration absorption peak, respectively. In addition, the bands at 1,720 and 1,090 cm^−1^ are attributed to C=O bending vibration and C-H bending vibration. The presence of above several absorption peaks are sufficient to prove the existence of PAA (Wang et al., 2015). The weaker absorption peak from 1,400 to 1,650 cm^−1^ belongs to the polyaniline. The two characteristic bands at 1,490 and 1,580 cm^−1^ are attributed to the stretching vibration of benzenoid C=C and the stretching vibration of quinonoid C=C, respectively. The band at 1,630 cm^−1^ can be assigned to N-H bending vibration (Li et al., 1999; Sivakkumar and Kim, 2007). The results confirm that the *in-suit* polymerization of PAA and aniline lead to the formation of PAA-PANi.

**Figure 3:**
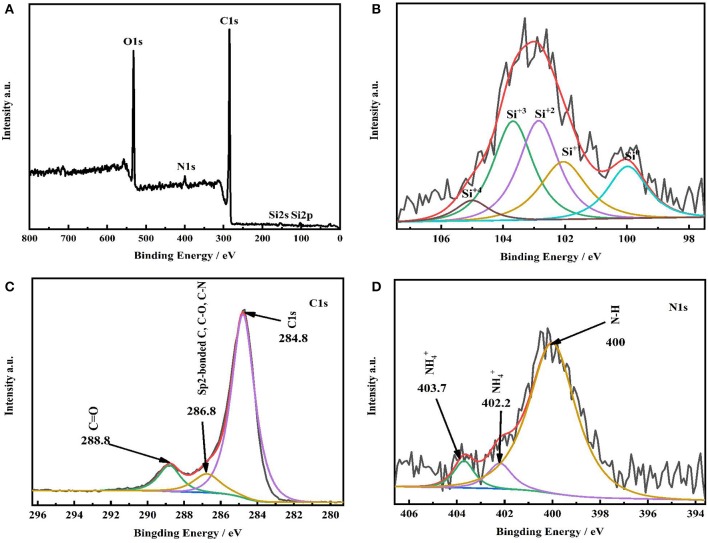
**(A–D)** XPS spectra of SiO_x_-G/PAA-PANi/graphene particles.

The element details of SiO_x_-G/PAA-PANi/graphene were reflected by XPS. Figure 3 shows the spectrum of all elements (Figure 3A), Si2p (Figure 3B), C1s (Figure 3C), and N1s (Figure 3D). The detail information about valence states of Si obtained from Figure 3B and described in Table 1. Clearly, the existence of Si^0^(99.98 eV), Si^1+^(102.06eV), Si^2+^(102.85eV), Si^3+^(103.68eV), and Si^4+^(105.00eV) can be observed in the SiO_x_-G/PAA-PANi/graphene sample, and the corresponding atomic percentages are 15.55, 20.11, 29.63, 29.63, and 5.08%, respectively (Zheng et al., 2018). The average valence of Si calculated from the Si 2p spectrum is 1.88. The XPS results of Figure 3B verifies the successful synthesis of SiO_x_ from the commercial SiO. The three peaks (Sp2-bonded C, C-O and C-N) deconvoluted from the C1s (Figure 3C) spectrum are situated at 286.75 eV, and C=O located at 288.8 eV. The bonding energy of C1s for graphite is located at 284.8 eV, indicating that graphite is present in SiO_x_-G/PAA-PANi/graphene. It can be obtained from the N1s spectrum (Figure 3D) that there is a strong peak at 400.00 eV, being in correspondence with the characteristic chemical N-H of PANi. The protonated amines are located at 402.20 and 403.70 eV. N1s spectrum (Figure 3D) and FT-IR (Figure 2) images further confirmed the successful preparation of PAA-PANi by *in-situ* polymerization.

**Table 1:** The proportion of different valence states of Si in SiO_x_-G/PAA-PANi/graphene.

**Production**	**Si^0^**	**Si^+1^**	**Si^+2^**	**Si^+3^**	**Si^+4^**
Position/eV	99.98	102.06	102.85	103.68	105.00
Percentage/%	15.55	20.11	29.63	29.63	5.08

**Figure 4:**
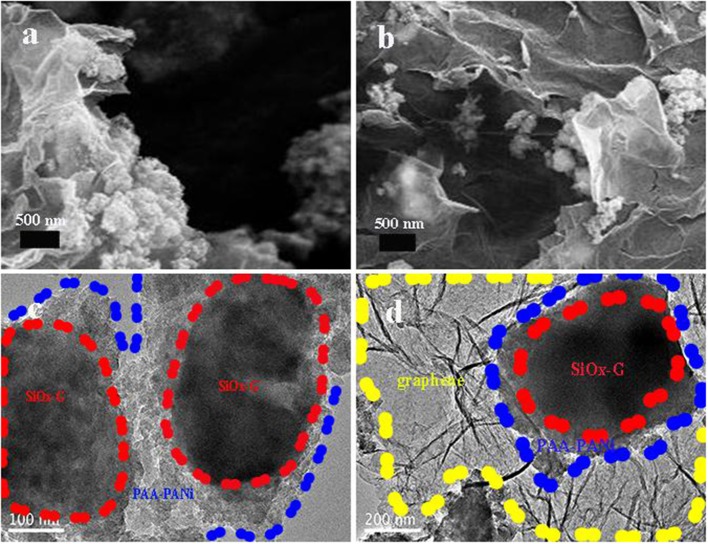
**(a,b)** SEM images of SiO_x_-G/PAA-PANi/graphene; **(c)** TEM image of SiO_x_-G/PAA-PANi; **(d)** TEM image of SiO_x_-G/PAA-PANi/graphene.

Figures 4a,b shows the SEM images of SiO_x_-G/PAA-PANi/graphene. As clearly seen, graphene is distributed around SiO_x_-G/PAA-PANi, which plays a supporting connection in the structure and can improve the conductivity. This results match with the results shown by the TEM (Figures 4c,d). Figure 4c shows the TEM image of SiO_x_-G/PAA-PANi. As obtained from Figure 4a, the SiO_x_-G particles are encapsulated inside the conductive hydrogel, and the coating thickness range of PAA-PANi ranges from 100 nm to 200 nm. Figure 4d depicts the TEM image of SiO_x_-G/PAA-PANi/graphene. It can be observed that SiO_x_-G/PAA-PANi particles are distributed between graphene.

**Figure 5:**
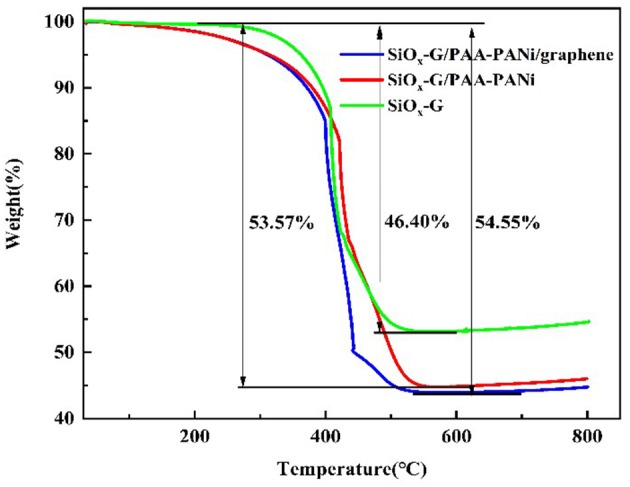
TG image of SiO_x_-G, SiO_x_-G/PAA-PANi and SiO_x_-G/PAA-PANi/graphene.

The estimate of the carbon contents based on the three samples was obtained via TG measurement. The carbon contents of SiO_x_-G, SiO_x_-G/PAA-PANi, and SiO_x_-G/PAA-PANi/graphene were about 46.85, 55.22, and 56.08 wt%, respectively (Figure 5). The superficial passivation of SiO_x_ lead to the oxidation of Si in air was not distinguished between 600 and 800°C (Chen et al., 2014; He et al., 2019; Jiang Y. et al., 2019; Jiang Z. et al., 2019). They are beneficial to improve the cycle performance of SiO to varying degrees.

**Figure 6:**
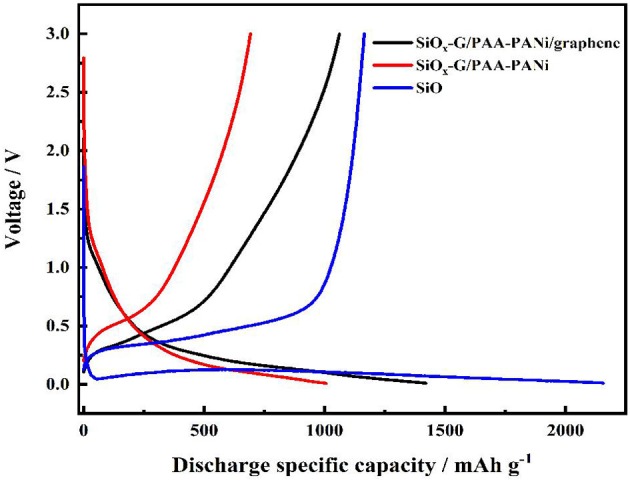
The charge/discharge curves of SiO_x_-G/PAA-PANi/graphene, SiO_x_-G/PAA-PANi, and SiO anodes under the current density of 500 mA g^−1^.

Figure 6 displays the charge/discharge curves for SiO_x_-G/PAA-PANi/graphene, SiO_x_-G/PAA-PANi and SiO at a current density of 100 mA g^−1^. The charge/discharge curves of the SiO electrode deliver a high initial discharge specific capacity of 2,156 mA h g^−1^ and an initial charge specific capacity of 1,165 mA h g^−1^ (ICE is 54%) and show a stable and obvious voltage platform around 0.1 V, in which the gentle slopes appears between 0.2 and 0.5 V. The first discharge-charge profiles of the SiO_x_-G/PAA-PANi and SiO_x_-G/PAA-PANi/graphene electrodes are similar to that of the Si-SiO_x_-Cristobalite/Graphite electrode (Ren and Li, 2014). The first discharge and charge capacities of SiO_x_-G/PAA-PANi composite are 1006.4 and 692.3 mA h g^−1^, respectively, with an ICE of about 68.8%. The capacity of the first discharge and charge capacity of SiO_x_-G/PAA-PANi/graphene is 1420.8 and 1062.3 mA h g^−1^, respectively, with an ICE of about 74.77%. The large initial irreversible capacity is ascribed to the Li^+^ consumption of to form the SEI film as well as the chemical reactions between Li and SiO_x_.

**Figure 7:**
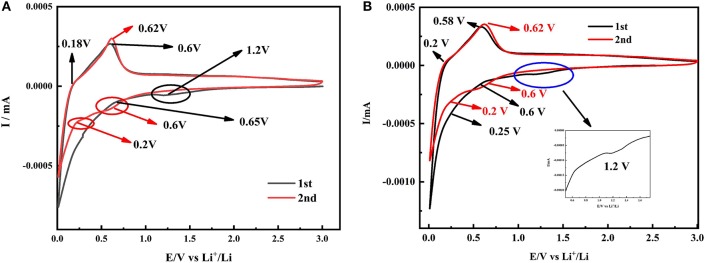
CV curves of SiO_x_-G/PAA-PANi **(A)** and SiO_x_-G/PAA-PANi/graphene **(B)** electrodes at the scanning rate of 0.1 mV s^−1^.

Figure 7A displays the cyclic voltammetry curves of the SiO_x_-G/PAA-PANi. A relatively flat reduction peak appearing at 1.2 V and a strong reduction peak appearing at 0.65 V in the first lithiation process are corresponding to the decomposition of liquid electrolytes and formation of the SEI film, respectively (Ren and Li, 2014). The oxidation peak appearing at 0.18 V indicates that Li is detached from Li_x_C, and the voltage position representing Li detached from Li_x_Si is at 0.6 V in the first delithiation (Sivakkumar and Kim, 2007; Chen et al., 2014, 2018; Li et al., 2019). The reason for the incomplete reversibility of the electrode capacity is attributed to the by-products (Li_2_O and Li_2_Si_2_O_5_) formed by the irreversible chemical reactions between SiO_x_ and Li (Sivakkumar and Kim, 2007). There are two reduction peaks around 0.6 V and 0.2 V, which represent the lithiation process. Figure 7B shows the cyclic voltammetry curves of the SiO_x_-G/PAA-PANi/graphene composite. Comparing with the CV curves of the SiO_x_-G/PAA-PANi, there are the same reduction peaks around 0.6 and 0.2 V in the first cycle of SiO_x_-G/PAA-PANi/graphene, which are attributed to lithium extraction from the SiO_x_-G/PAA-PANi/graphene.

**Figure 8:**
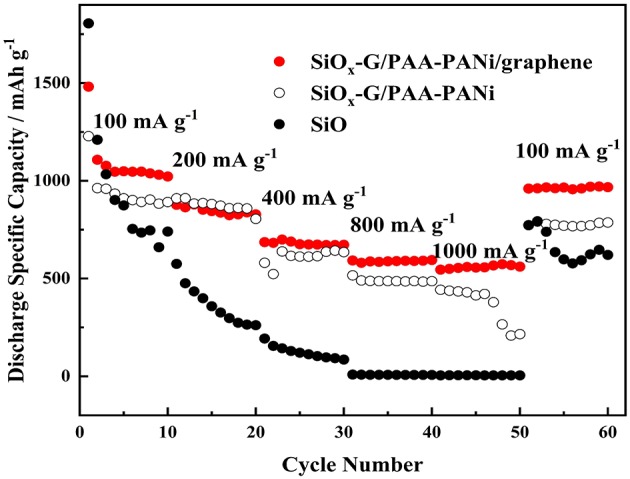
The rate performances of SiO_x_-G/PAA-PANi/graphene, SiO_x_-G/PAA-PANi, and SiO electrodes.

The rate performance of the SiO_x_-G/PAA-PANi/graphene, SiO_x_-G/PAA-PANi, and SiO electrodes is illustrated in Figure 8. The rate performance of the SiO electrode is significantly different from that of the SiO_x_-G/PAA-PANi electrode, in which the rate performance of the SiO electrode is so unsatisfactory. Based on these results, the doping of conductive graphene further enhances the rate performance. The capacities of the SiO_x_-G/PAA-PANi/graphene are 1022.3, 828.1, 675.7, 588.1, 556.2 mA h g^−1^ at a current density of 100, 200, 400, 800, and 1,000 mA g^−1^, respectively. Moreover, the capacity can rise to 967.1 mA h g^−1^ as the current density returns to 100 mA g^−1^, which demonstrates that SiO_x_-G/PAA-PANi/graphene electrode can maintain a good structural stability.

**Figure 9:**
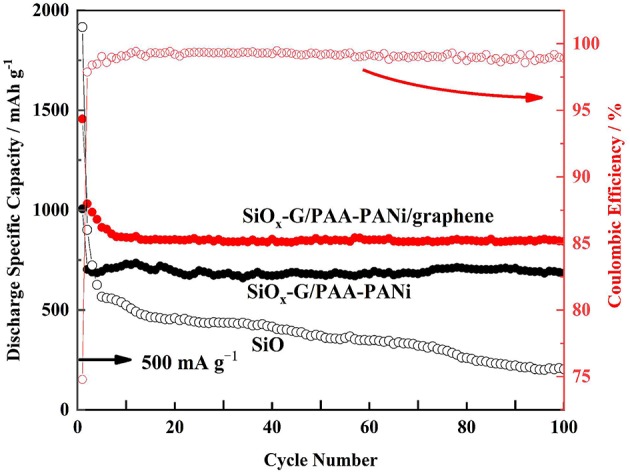
Long-time cycling performances (discharge capacities) of SiO_x_-G/PAA-PANi/graphene, SiO_x_-G/PAA-PANi, and SiO under the current density of 500 mA g^−1^.

Figure 9 indicates the cycling stability of SiO_x_-G/PAA-PANi/graphene, SiO_x_-G/PAA-PANi, and SiO electrodes at a current density of 500 mA g^−1^. Although the initial discharge specific capacity of the SiO electrode reaches 1916.6 mA h g^−1^, the capacity suddenly drops due to the rupture of the SiO structure. Compared with SiO, the cycle performance of SiO_x_-G/PAA-PANi is significantly improved, and a high discharge specific capacity (685.4 mA h g^−1^) is maintained when it is cycled to the 100th cycle, which can be attributed to the 3D structure of the conductive hydrogel that can serve as an effective buffer for the volume change of SiO_x_ nanoparticles. In contrast to SiO_x_-G/PAA-PANi, SiO_x_-G/PAA-PANi/graphene exhibits a significantly improved performance, with a discharge specific capacity of 842.3 mA h g^−1^ at current density of 500 mA g^−1^ at 100th cycle, and the coulombic efficiency is about 99% from 8th to 100th cycles, which is due to the improved electron transport.

## Conclusions

In summary, the three-dimensional SiO_x_-G/PAA-PANi/graphene hydrogel was prepared by a facile ball milling and *in-situ* polymerization process. The amorphous SiO_x_-G was encapsulated within 3D mesh structure of PAA-PANi/graphene. According to the preparation method of the study, the specific capacity of SiO_x_-G/PAA-PANi/graphene can be as high as 842.3 mA h g^−1^ at 500 mA g^−1^ when Li^+^ are subjected to the 100th deintercalation, as well as the ICE is increased to 74.77% compared with SiO. The superior performance could be due to that PAA-PANi offers fast channels for electronic and ionic transfer and free space for SiO_x_-G volume changes, and also the conductively active graphene effectively improves the electron transport.

## Data Availability Statement

The raw data supporting the conclusions of this article will be made available by the authors, without undue reservation, to any qualified researcher.

## Author Contributions

YR contributed conception, design of the study, and revised the manuscript. YL carried out experiments and wrote the manuscript. KL performed analyzed experimental results. XH revised the manuscript.

### Conflict of Interest

The authors declare that the research was conducted in the absence of any commercial or financial relationships that could be construed as a potential conflict of interest.
